# Traditional serrated adenoma with superficially serrated adenoma: A case treated with endoscopic submucosal dissection with detailed endoscopic observation and pathological study

**DOI:** 10.1002/deo2.247

**Published:** 2023-06-01

**Authors:** Momoka Sato, Tomoji Kato, Yu Tezen, Takeshi Hayashi, Toru Matsui, Katsuya Kobayashi, Kenichiro Sekigawa, Ryosuke Tajiri, Hiroshi Mitsui

**Affiliations:** ^1^ Department of Gastroenterology Tokyo Teishin Hospital Tokyo Japan; ^2^ Department of Pathology Tokyo Teishin Hospital Tokyo Japan

**Keywords:** endoscopic submucosal dissection, *KRAS*, RSPO, superficially serrated adenoma, traditional serrated adenoma

## Abstract

A new subtype of serrated lesions, superficially serrated adenoma (SuSA), has been proposed as a lesion that histopathologically exhibits the morphological features of both conventional adenomas and serrated lesions and is difficult to classify as either one. SuSA has been elucidated to be a precursor lesion of *KRAS‐*type traditional serrated adenoma. It has also been reported that SuSA may have malignant potential.

We report a case treated with endoscopic submucosal dissection and detailed observation. Endoscopy revealed a raised lesion with a two‐tier raised appearance in the sigmoid colon: a tall pinecone‐like reddish structure and flattened whitish elevation on white light imaging. Magnifying narrow‐band imaging revealed conspicuous blood vessels in the pinecone‐like structure and slightly dilated reticular vessels in the flattened area. Crystal violet staining showed that the pinecone‐like structure had a type IV_H_ pit pattern and the flattened area had a stellate to slightly elongated type III_H_ pit pattern diagnosed based on Kudo's classification and other pit pattern classification systems. Ki67‐positive cells were distributed in the basal and middle layers of the gland in the flattened elevated area. Genetic analysis results were positive for *KRAS* mutation and negative for *BRAF* mutation. Histopathological examination revealed a traditional serrated adenoma in the pinecone‐like structure and SuSA in the adjacent flattened elevated area.

## INTRODUCTION

Superficially serrated adenoma (SuSA) was proposed as a new subtype of serrated lesions by Hashimoto et al.^1^ It is considered common in the sigmoid colon and rectum. Histopathologically, SuSA has morphological features of both conventional‐type adenomas and serrated lesions, making it difficult to classify the lesions as either one. Since SuSA has been elucidated to be a precursor lesion of *KRAS*‐subtype traditional serrated adenoma (TSA), it may have malignant potential.^2^ However, in today's colonoscopy practice, SuSA may be misdiagnosed as hyperplastic polyps with no malignant potentials and left without removal.

To date, there are still few reports on the clinicopathologic features and detailed endoscopic findings of SuSA. Herein, we report a case of TSA with SuSA carefully detected by colonoscopy and treated with endoscopic submucosal dissection, wherein an immunohistochemistry analysis was performed.

## CASE REPORT

**FIGURE 1:**
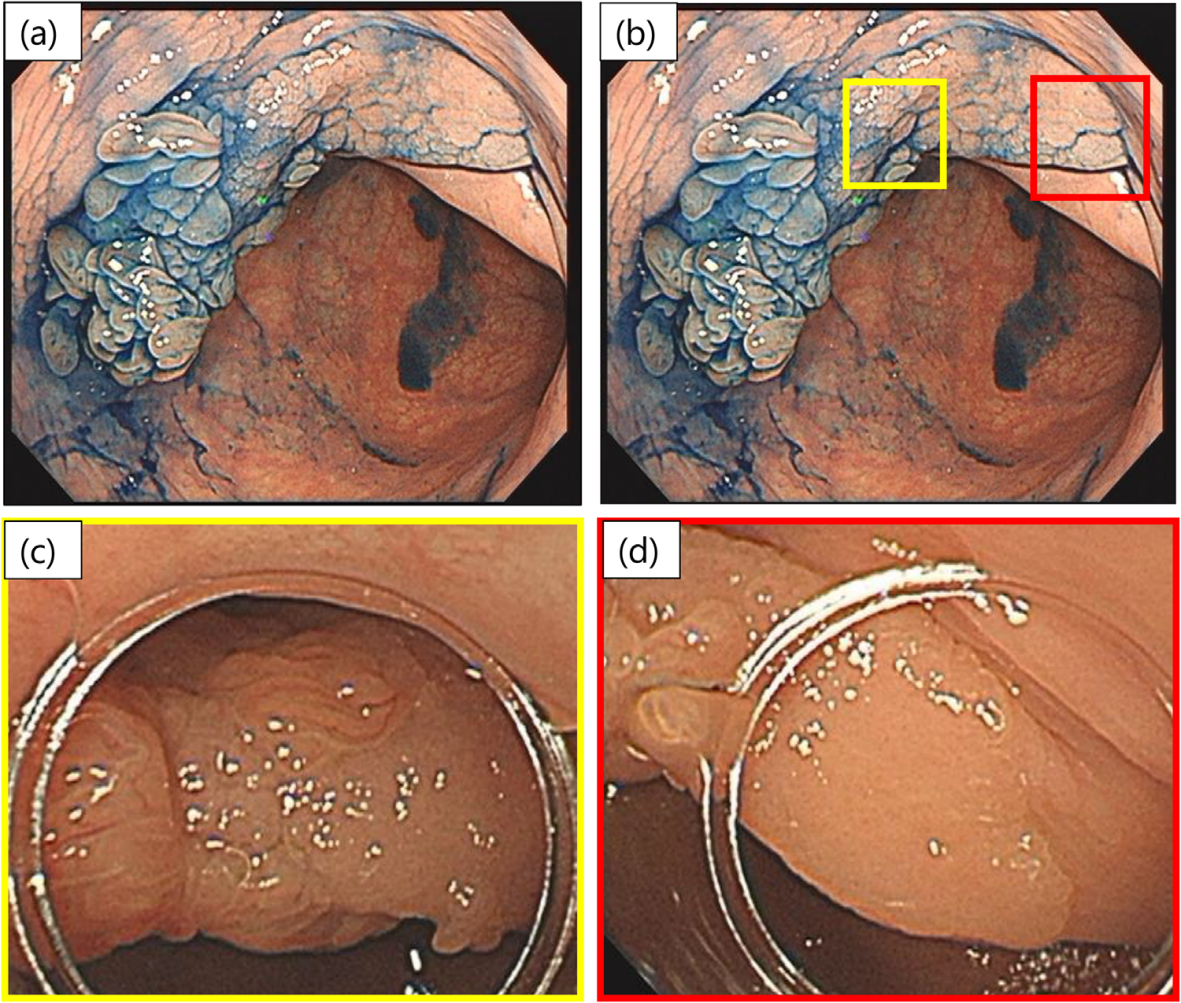
Endoscopic findings of traditional serrated adenoma (TSA) with superficially serrated adenomas (SuSA). (a) Colonoscopy shows a raised lesion with a two‐tier raised appearance in the sigmoid colon, consisting of a tall pinecone structure and flattened elevation. Indigo carmine dye spray highlights the nodular surface. (b) The yellow box indicates the boundary between the TSA and SuSA components, which is magnified in Figure 1c. The red box indicates a typical part of the SuSA component, which is magnified in Figure 1d. (c) High‐power view showing the boundary between the TSA and SuSA components (the yellow box in Figure 1b). The TSA component appears reddish on white‐light imaging. (d) High‐power view of the SuSA component (the red box in Figure 1b). The SuSA component appears white, with a smooth surface on white‐light imaging.

A 74‐year‐old woman was referred to our hospital for endoscopic resection of a polyp in the sigmoid colon. Colonoscopy showed a raised lesion, approximately 25 mm in size, with a two‐tier raised appearance in the sigmoid colon, consisting of a tall pinecone‐like structure and a flattened elevation (Figure 1a). The pinecone‐like structure appeared reddish (Figure 1c), and the flattened area was white to the same color as the surrounding background mucosa (Figure 1d) on white‐light imaging.

**FIGURE 2:**
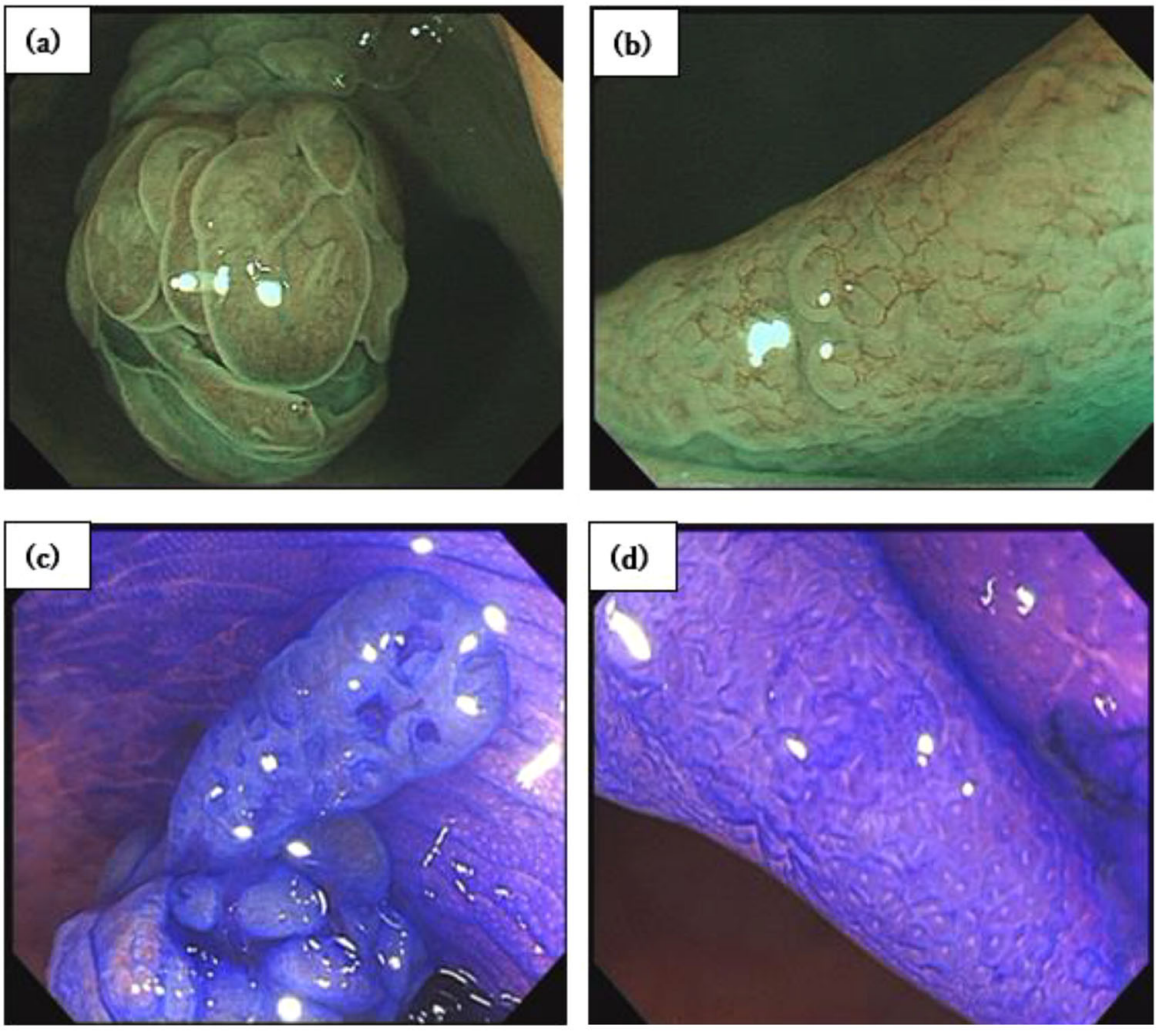
Narrow‐band imaging (NBI) and crystal violet staining revealing features of traditional serrated adenoma (TSA) and superficially serrated adenoma (SuSA). (a) NBI magnification reveals conspicuous blood vessels in the pinecone structure of the TSA. (b) The vessels are unclear on magnifying NBI in the flat areas of the SuSA. (c) Crystal violet staining shows that the pinecone structure of the TSA has a type IV_H_ pit pattern. (d) Crystal violet staining shows that the flat area of the SuSA has a stellate to slightly elongated type III_H_ pit. Abbreviation: NBI, narrow‐band imaging.

Magnifying narrow‐band imaging (NBI) revealed conspicuous blood vessels in the pinecone‐like structure (Figure 2a) and slightly dilated reticular vessels in the flattened areas (Figure 2b). Crystal violet staining showed that the pinecone‐like structure had an IV_H_‐type pit pattern, diagnosed based on Kudo's classification^3^ and other pit pattern classification systems^4^, ^5^, ^6^ (Figure 2c). The flattened area showed a stellate to slightly elongated III_H_‐type pit pattern (Figure 2d).

Based on these findings, endoscopic submucosal dissection was performed, considering the TSA in the pinecone‐like structure and adenoma or SuSA in the adjacent flattened structure. The patient underwent an en‐bloc resection without any adverse events.

**FIGURE 3:**
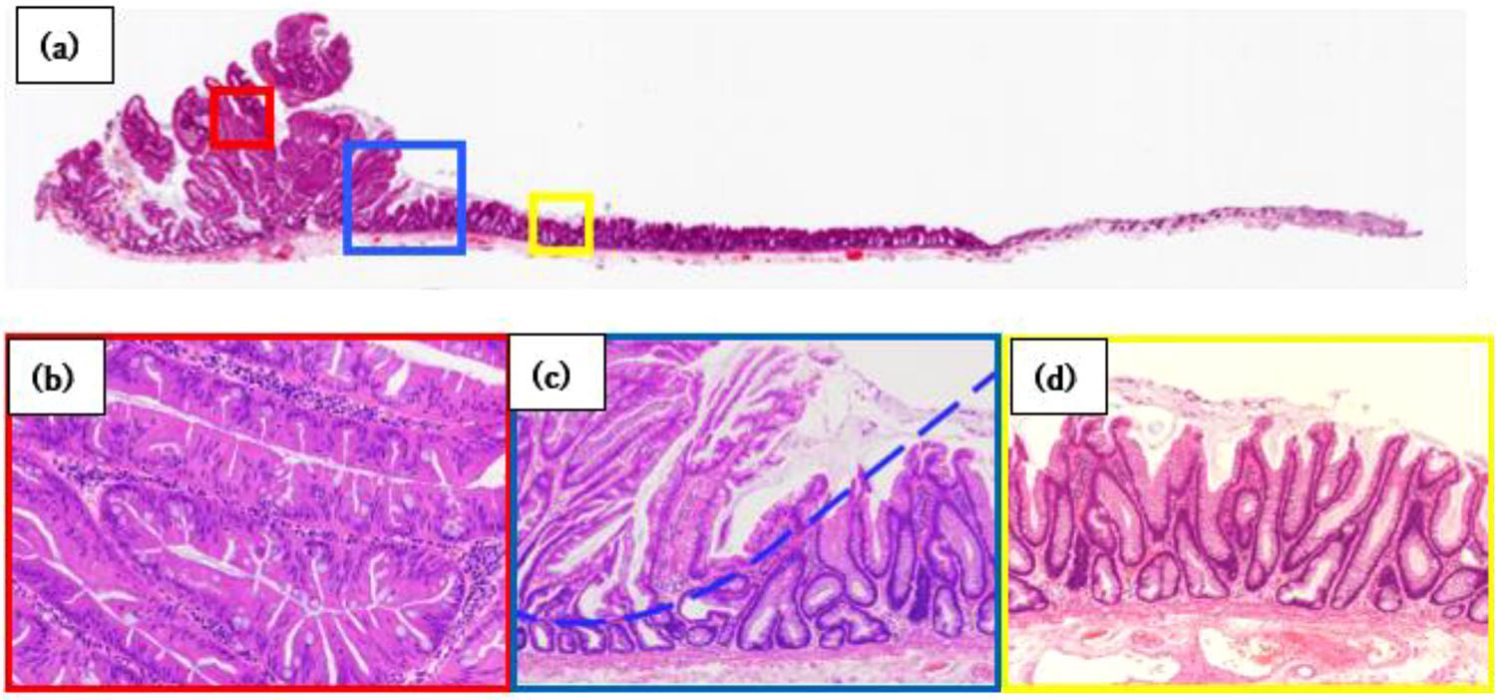
Histological features of traditional serrated adenoma (TSA) with superficially serrated adenomas (SuSA). (a) Low‐power images of the polyps showing the proliferation of adenomatous glands and superficial serration using H&E staining. (b) High‐power view of the TSA component (the red box in Figure 3a). The TSA component shows classical histological features, such as villous architecture and ectopic crypt formation. (c) High‐power view showing the boundary between TSA and SuSA components. A clear demarcation line (blue broken line) can be drawn between the TSA and SuSA (the blue box in Figure 3a). (d) High‐power view showing the SuSA (the yellow box in Figure 3a). The SuSA component consists of straight serrated adenomatous glands exhibiting serration confined to the superficial layer. Serration is confined to the surface epithelium and crypt orifice. Histopathological examination reveals a dense proliferation of glandular ducts with pseudostratified nuclei and a serrated surface around the TSA. Slit‐like serrations, penicillate nuclei, and ectopic crypt foci are observed in the pinecone structure. These are specific features of the SuSA. Abbreviation: HE, hematoxylin and eosin.

Histopathological examination revealed a dense proliferation of the glandular ducts with pseudostratified nuclei and a serrated surface around the TSA lesion, forming tall ridges (Figure 3a).

**FIGURE 4:**
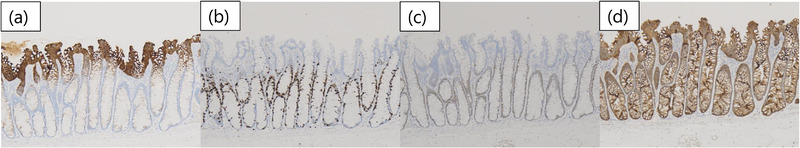
Immunohistochemical staining of superficially serrated adenoma (SuSA). (a) CK20 is expressed in the upper layer of the SuSA. (b) Ki‐67 expression is distributed in the middle to lower layers of the SuSA. (c) C‐MYC expression is distributed in the middle to lower layers of the SuSA. (d) A SuSA shows moderate nuclear β‐catenin staining in addition to membranous staining.

Immunohistochemical staining showed that CK20 was expressed in the upper layer of the flattened structure adjacent to the TSA lesion (Figure 4a).

Ki‐67 and MYC expressions were distributed in the basal and middle layers of the flattened structure (Figure 4b,c). Additionally, β‐catenin, suggesting the activation of the Wnt pathway, was found in some nuclei (Figure 4d). These are specific features of the SuSA.

Genetic analysis revealed positive results for *KRAS* mutation and negative for *BRAF* mutation. No tests could be performed for *RSPO* fusions. The histopathological findings and *KRAS* mutation strongly suggested the possibility of TSA with SuSA.

## DISCUSSION

In 2018, a new subtype of serrated lesions, SuSA, has been proposed as a new subtype of serrated lesions that manifests the morphological features of both conventional adenomas and serrated lesions and is difficult to classify as either one.^1^ It is known that some types of SuSA are associated with TSA.

In a report examining 21 cases of TSA with SuSA by Mizuguchi et al., the median age was 67 years, of which the majority was female. All lesions were located in the sigmoid colon or rectum and had a median diameter of 15 mm. Genetic analysis results showed *RSPO* mutation/overexpression in 95% of both TSA and SuSA components and *KRAS* mutations in all patients.^7^

Endoscopic findings revealed a two‐tier raised elevation, with a flat portion (SuSA) and a taller elevated portion (TSA). The SuSA portion appeared white, whereas the TSA portion appeared reddish on white‐light imaging.

Although the NBI findings clearly showed blood vessels in the TSA area, none were observed in most SuSA areas. Chromoendoscopy revealed a type III_H_ pit pattern in the SuSA areas.

Tanaka et al. studied the histopathology of TSA and its associated lesions according to *KRAS* and *BRAF* mutations. They found that all TSA with associated lesions showing *KRAS* mutations were located in the distal colon. These lesions were considered SuSA‐like lesions and reported to show a type II_L_ pit pattern in the associated portion without endoscopic mucus signs or II‐Open pit pattern.^8^ Chino et al. reported a group of lesions with serrated, adenomatous structures with TSA that were difficult to classify based on the criteria for conventional diagnosis as tubuloserrated adenomas.

All lesions in previous reports were located in the distal colon and had sessile‐elevated central components resembling mixed adenomatous morphology and included various pit patterns such as typeIV with serration, type II, and type III_L_. All these lesions had *KRAS* mutations without *BRAF* ones and were reported to be similar to SuSAs.^9^ Since genetic analysis showed *RSPO* fusion/overexpression at a high rate in both TSA and SuSAs in these reports, TSA presenting with a two‐stage elevation in the distal colon with *KRAS* mutations may be considered associated with SuSAs.

In our case, the flattened component of the two‐stage elevated lesion, the SuSA, showed a type II pit pattern, a stellate cap with an elongated type II pit pattern, and a partially branching pit pattern. In addition, the flattened component showed a network pattern of vessels on NBI.

Although vascular structures have not been reportedly recognized in approximately 80% of SuSA lesions, vessels could be seen in our case, and the caliber of the vessels in the flattened component was similar to that in the surrounding normal mucosa. Therefore, this lesion was diagnosed as similar to type I according to the Japan NBI Expert Team classification.^10^

Genetic analysis showed *KRAS* mutations in both lesions, without performing analysis for *RSPO* fusions, as the *RSPO* gene fusion test could only be performed in a limited number of facilities.

Since the SuSA may not only be a precursor lesion of TSA but also have malignant potential,^2^ colonoscopy should be carefully performed for detecting spreading tumors around TSA lesions, particularly when these TSA lesions are noticed in the distal colon. Further studies are required to determine the clinical features of this condition.

## CONFLICT OF INTEREST STATEMENT

None.
